# Encoding Manual Dexterity through Modulation of Intrinsic α Band Connectivity

**DOI:** 10.1523/JNEUROSCI.1766-23.2024

**Published:** 2024-03-27

**Authors:** Ottavia Maddaluno, Stefania Della Penna, Alessandra Pizzuti, Matteo Spezialetti, Maurizio Corbetta, Francesco de Pasquale, Viviana Betti

**Affiliations:** ^1^Department of Psychology, Sapienza University of Rome, Rome 00185, Italy; ^2^IRCCS Santa Lucia Foundation, Rome 00179, Italy; ^3^Department of Neuroscience, Imaging and Clinical Sciences and ITAB - Institute of Advanced Biomedical Technologies, “G. d’Annunzio” University of Chieti and Pescara, Chieti 66013, Italy; ^4^Department of Neuroscience and Padova Neuroscience Center, University of Padua, Padua 35131, Italy; ^5^Veneto Institute of Molecular Medicine (VIMM), Padova 35129, Italy; ^6^Faculty of Veterinary Medicine, University of Teramo, Teramo 64100, Italy

**Keywords:** functional connectivity, human behavior, magnetoencephalography, manual dexterity, motor tasks, resting state

## Abstract

The human hand possesses both consolidated motor skills and remarkable flexibility in adapting to ongoing task demands. However, the underlying mechanisms by which the brain balances stability and flexibility remain unknown. In the absence of external input or behavior, spontaneous (intrinsic) brain connectivity is thought to represent a prior of stored memories. In this study, we investigated how manual dexterity modulates spontaneous functional connectivity in the motor cortex during hand movement. Using magnetoencephalography, in 47 human participants (both sexes), we examined connectivity modulations in the α and β frequency bands at rest and during two motor tasks (i.e., finger tapping or toe squeezing). The flexibility and stability of such modulations allowed us to identify two groups of participants with different levels of performance (high and low performers) on the nine-hole peg test, a test of manual dexterity. In the α band, participants with higher manual dexterity showed distributed decreases of connectivity, specifically in the motor cortex, increased segregation, and reduced nodal centrality. Participants with lower manual dexterity showed an opposite pattern. Notably, these patterns from the brain to behavior are mirrored by results from behavior to the brain. Indeed, when participants were divided using the median split of the dexterity score, we found the same connectivity patterns. In summary, this experiment shows that a long-term motor skill—manual dexterity—influences the way the motor systems respond during movements.

## Significance Statement

Using hands efficiently is central to our daily life. However, individuals differ in manual dexterity. We study whether the brain's functional organization encodes variability in manual behavior. Using a large set of MEG data acquired during rest and a finger tapping task, we investigated how hand movements change the intrinsic functional connectivity and network architecture. Specifically in the α band, we demonstrate that higher dexterity is associated with decreased connectivity, increased segregation, and reduced nodal centrality. Low dexterous individuals show opposite patterns. We concluded that manual dexterity influences how the motor system responds during movements. These findings yield high potential to understand how intrinsic connectivity retains relevant behavior and to develop neural biomarkers of pathological behavior.

## Introduction

Healthy individuals differ in their manual dexterity. This ability is fundamental to efficiently interact with the environment. There is evidence that manual dexterity correlates with structural and functional changes in the motor cortex. For instance, keyboard and string players have larger primary motor cortex (Amunts et al., 1997), gray matter density (Gaser and Schlaug, 2003; Han et al., 2009), and an extension of the sensory representation of the digits (Elbert et al., 1995; Elbert and Rockstroh, 2004) than nonexpert individuals. Furthermore, the anterior corpus callosum is larger in experts than that in naive (Schlaug et al., 1995), likely representing a morphological substrate of increased interhemispheric communication subserving hand motor sequences. Finally, motor experts activate the motor cortex more focally and efficiently (Pascual-Leone et al., 1995; Krings et al., 2000). However, less is known about how the functional organization of the brain correlates with long-term motor skills.

One leading hypothesis is that brain networks at rest (denoted resting state networks, RSNs) are sculpted over the lifespan of an individual by previous experiences and learning (Albert et al., 2009; Lewis et al., 2009; Ma et al., 2011; Taubert et al., 2011). Specifically, RSNs may represent a mechanism for storing and keeping online behaviorally relevant representations (Pezzulo et al., 2021). However, to our knowledge, no study to date has shown that long-term motor skills are already encoded in task-evoked changes of intrinsic activity and that such changes influence oncoming behavior.

Intrinsic activity is both stable and flexible. Its topography is stable across participants and recording sessions (Damoiseaux et al., 2006) and resilient across behavioral states and levels of consciousness (Greicius et al., 2008; Betti et al., 2013, 2018; Cole et al., 2014; Krienen et al., 2014; Spadone et al., 2015). On the other hand, performing a task modifies the strength of the intrinsic functional connectivity (Lewis et al., 2009; Tambini et al., 2010; Betti et al., 2013, 2018; Harmelech and Malach, 2013; Cole et al., 2014; Spadone et al., 2015), and these changes can last for over a week after learning (Taubert et al., 2011). This malleability meets the requirement of flexibility. Flexibility means that connections that are highly correlated during resting state change their strength, frequency content, or topological organization during a motor task. Stability makes the functional organization between rest and task performance highly similar. In this study, we tested the hypothesis that the differences in dexterity may rely upon patterns of stability and flexibility of neural communication, especially within the motor system.

Flexibility is a fundamental mechanism that allows adaptive behavior and learning (Bassett et al., 2011). In terms of brain connectivity, it requires both segregation (i.e., independent processing in specialized networks) and integration (i.e., communication among networks; Bassett et al., 2011, 2015; Favaretto et al., 2021). Crucially, a dynamic balance of segregation and integration is critical for normal behavior (Fornito et al., 2012; Shine et al., 2016). In addition, learning (e.g., motor learning (Bassett et al., 2015) can modulate the underlying topology, for example, modularity (decomposability into distinct partitions of the network) and hub centrality (the role of highly interconnected regions) of the brain.

Here, we study how brain networks measured with magnetoencephalography (MEG) in healthy volunteers change from a rest condition to performing motor tasks (hand or foot movements). Brain networks are characterized in α and β bands both in terms of large-scale connectivity (e.g., strength, frequency content) and architecture (i.e., integration/segregation). Then, we employ a data-driven analysis to characterize the individual variability of MEG connectivity changes and identify two groups of subjects. Finally, we relate these different connectivity profiles to manual dexterity measured on a speeded visuomotor reaction time task (nine-hole peg test). Specifically in the α band, we find that individuals with higher dexterity show increased segregation/modularity and decreased nodal centrality when going from rest to movement, especially in the motor cortex and dorsal attention network (DAN). Opposite patterns were found in individuals with low dexterity. These results show that pre-existent manual ability influences the way the motor cortex reorganizes during ongoing task demands.

## Materials and Methods

### Participants

We analyzed MEG data from 47 participants (mean age ± SD = 27.8 ± 3.8 years, 25 females, 22 males), a subset of the freely available data collected as part of the Human Connectome Project release (HCP S1200 Release, WU-Minn HCP Consortium). Among the available HCP data, we considered right-handed participants (mean handedness score = 79.04 ± 18.76) who have both rest and task blocks and met the minimal criteria for data quality (see below). HCP data were acquired using protocols approved by the Washington University institutional review board. Informed consent was obtained from all subjects. Anonymized data are publicly available from ConnectomeDB (https://db.humanconnectome.org).

### Experimental design and statistical analysis

**Figure 1.**
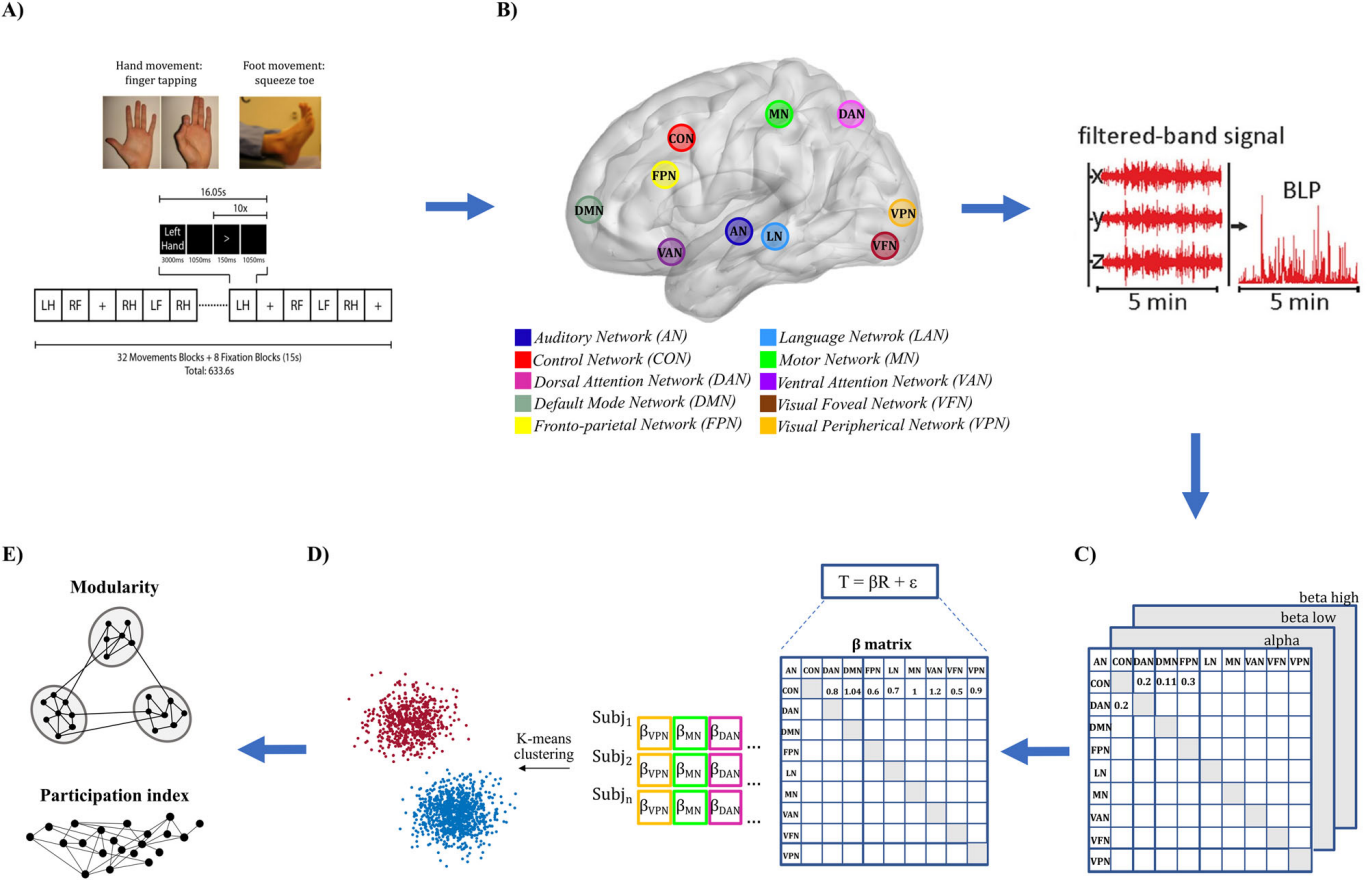
Experimental paradigm and analysis pipeline. ***A***, Participants performed a finger tapping (with their right or left hand) or a toe squeezing (with their right or left foot). The motor task consisted of 32 movement blocks (16 hand and 16 foot). Each block consists of 10 trials. Three resting state runs lasting 6 min each precede the motor task. ***B***, A set of 164-node brain parcellation, comprising 10 networks, is used to estimate the BLP in the α (*α*, 8–15 Hz), low β (β, 15–26 Hz), and high β (26–35 Hz) band. ***C***, The static functional connectivity is calculated as the leakage-corrected correlation between each pair of nodes, separately for the resting state and the task data. ***D***, Linear model for the relationship between rest and task. A *K*-means algorithm on the *β* values of the model of each subject identifies two groups (i.e., high and low performers, blue and red, respectively). ***E***, Measures of segregation/integration are computed.

MEG data were acquired with a MAGNES 3600 scanner system with 248 channels (4D Neuroimaging), at a sampling rate of 2,034.5 Hz. Subjects were first recorded during three blocks of visual fixation (rest), each lasting 6 min, and then during two runs of three types of tasks, the latter of which consisted of a motor task lasting 14 min (Larson-Prior et al., 2013). The motor task started after an approximately 10 min break, during which EMG electrodes were mounted. During the motor task, participants were presented with visual cues providing instructions about the movement to perform with their right or left hand or right and left foot. The hand movements consisted of a finger tapping task involving the thumb and the index finger, whereas for the foot condition, they performed toe squeezing. The design of the motor task (Fig. 1*A*) included 32 movement blocks, 8 for each hand and foot lasting 12 s and 10 interleaved fixation blocks lasting 15 s. Each movement block was composed of a 3 s cue suggesting the next movement for participants to perform followed by a 1,050 ms black screen period. Then 10 repetitions of visual pacing stimuli lasting 150 ms indicated the beginning of the movement followed by a 1,050 ms black screen period in which the movement should be performed. MEG data were recorded together with four electromyography (EMG) channels, placed on each hand and foot, two electrooculography (EOG) channels, and one electrocardiography (ECG) channel. The HCP database also provides preprocessed individual anatomical models computed from structural MRI, necessary for source reconstruction. We used the nine-hole peg test scores (provided by the HCP) for assessing manual dexterity (Gershon et al., 2010). The dexterity scores consist of the time in seconds employed to perform the test with the dominant hand. Notably, such a test is a simple and low-cost measure with high test–retest reliability (Wang et al., 2015), as recently shown also on a sample of HCP participants (Ruck and Schoenemann, 2021), thus representing a stable and long-term individual trait.

#### MEG data preprocessing and BLP estimation

MEG data released under the WU-Minn HCP project include unprocessed channel-level signals and channel-level preprocessed and source-level processed functional data, together with individual anatomical data. Here, we used channel-level preprocessed resting state data (Larson-Prior et al., 2013). Data in the unprocessed format were used for the motor task or when the processed data still contained artifacts. For these data, we applied the same preprocessing pipeline which produced the resting state data, to allow a reliable comparison among conditions. A brief description of the preprocessing pipeline is reported in the following. As a first step, data were bandpassed (1.3–150 Hz) and notch-filtered (59–61/119–121 Hz). Then, channels and signal segments contaminated by large artifacts (i.e., excess residual noise in the shielded room or muscular artifacts) were automatically identified and removed from further analysis. Specifically, noisy channels were identified through the low signal similarity with neighbors and measured through correlation and variance ratio and through the deviation from the distribution of channel weights obtained by an independent component analysis (ICA)–based approach (using FastICA with deflation approach). Then, we applied again the same ICA approach to sensor space MEG signals to identify environmental, physiological (e.g., cardiac, ocular), and residual channel artifacts and brain-independent components (brain ICs) from sensor space MEG signals, as previously reported (de Pasquale et al., 2010; Mantini et al., 2011; Betti et al., 2013). ICA separation was applied 20 times from different initial conditions. The ICs were automatically classified as brain or artifact for each of these iterations. For the classification, we used six parameters: (1) correlation of the IC time course with those of EOG and ECG channels filtered as the MEG data; (2) correlation of the IC power spectral density (PSD) with those of EOG and ECG channels; (3) correlation of the IC power time course with those of EOG and ECG channels; (4) temporal kurtosis; (5) 1/f trend of the IC PSD; and (6) flatness of the IC PSD. If none of these parameters was above the related thresholds (which are set within the HCP script after an ROC analysis over a set of independent HCP runs), then the IC was flagged as brain IC. The decomposition resulting in the highest number of brain ICs and the lowest level of artifact residual contamination (measured through the average (across the ICs) correlation between the brain ICs and the EOG/ECG channels) was retained as the best iteration and considered for successive steps. In our analysis, the same operator visually inspected the classification of the best iteration for resting state and task data, before proceeding.

The sensor maps of the brain IC were scaled to norm 100 and then projected in the source space by means of a Tikhonov-regularized minimum-norm estimator. The source space consisted of the individual surface-registered cortical sheet comprising 8,004 vertices. The noise level used by the WMNLS estimator was set to 8% of the maximum weight amplitude for each IC. We limited the analysis to a subset of the original 8,004 vertices, comprising functionally relevant nodes. Specifically, we considered the parcellation of 164 regions of interest (ROIs), consisting of vertices belonging to 10 networks depicted in Figure 1*B* (de Pasquale et al., 2021). We then applied a leakage-correction approach to the IC source space maps. Leakage is inevitable due to the application of projection schemes to solve the ill-posed inverse problem in MEG. Leakage typically yields a spatially blurred representation of the underlying source distribution. Thus, source space leakage effects lead to the spurious codependence of reconstructed sources, which heavily affects connectivity analysis. Hence, before estimating the functional connectivity, we applied the geometric correction scheme (GCS; Wens et al., 2015) as previously reported (Betti et al., 2018), where all the related formulas are reported. The GCS is a pairwise approach, that is, it models and removes the leakage spreading from a source vertex toward all other vertices based on the forward and inverse models. For each seed source, the vector activity of all the other vertices in the set was estimated as the linear combination of the brain IC time courses multiplied by the related leakage-corrected source space weights. For both experimental conditions, we then estimated the band-limited power (BLP) time courses as the mean of the activity square module over a sliding window lasting 400 ms, with a sampling rate equal to 50 Hz.

We restricted our analysis to the α (8–15 Hz), low β (15–26 Hz), and high β (26–35 Hz) bands, filtering the vector activity using separate high-pass and low-pass Butterworth filters. This choice was driven by previous studies showing that these frequency bands represented the neurophysiological correlates of RSNs (de Pasquale et al., 2010, 2012; Betti et al., 2013; Betti et al., 2018). The definition of the specific frequency ranges was based on the HCP manual and pipelines. Since the individual α peak occurred at frequency values larger than 10 Hz, the limits of this interval corresponded to the −3 dB value of the adopted high- and low-pass filters. This is also in line with previous literature for networks different from the MN (Spadone et al., 2021).

For the motor task only, we removed the evoked activity before BLP estimation, as in the following. For each direction of the vector activity of each vertex, we first averaged signals over epochs lasting 800 ms. The epoch onsets corresponded to the EMG trigger stored in the HCP database. We then applied the Gram–Schmidt orthogonalization approach as previously reported (Della Penna et al., 2004), retaining the residual signal for the BLP time course estimation.

#### Estimation of BLP functional connectivity

The static functional connectivity between a couple of nodes *i* and *j* was assessed as follows:

$$\begin{array}{ccc} \mathrm{FC}(i,j)=\mathrm{NaN}\;\mathrm{if}\;d(i,j)\leq 35\;\mathrm{mm}\\ \mathrm{otherwise} & & \\ \mathrm{FC}(i,j)=\left\{\frac{\mathrm{corr}(\mathrm{BLP}(i,i),\mathrm{BLP}(\;j,i))+\mathrm{corr}(\mathrm{BLP}(\;j,j),\mathrm{BLP}(i,j))}{2}\right\} \end{array}\;$$

where corr(*x*,*y*) is the Pearson's correlation coefficient between signals *x* and *y*, *d_ij_* is the Euclidean distance between ROIs *i* and *j*, and NaN is a “not a number” element for masking correlation coefficients closer than 35 mm. This mask was applied because GCS could be affected by local mis-correction effects, due to seed mislocalization (Wens et al., 2015). The element BLP (*i,j*) represents the BLP of ROI *i* after removing the bias due to the leakage spread from ROI *j*, and the diagonal element BLP(*i,i*) represents the uncorrected BLP of node *i*. We computed the average of the two pairwise correlations to account for slight asymmetries induced by possible numerical errors.

For each run of rest data, the static connectivity was computed over the entire session as the average of Pearson's correlation over nonoverlapping windows lasting 25 s. For the motor task, the static connectivity was computed over 12 s BLP concatenated segments belonging to the same class of movements. After averaging the interaction matrices across runs, we obtained for each experimental condition individual- and group-level static correlation matrices (Fig. 1*C*). To normalize the data, we computed the *z*-fisher transform of the correlation data at the subject level.

#### Analysis of network architecture

To investigate putative changes of the global functional architecture induced by switching from rest to the two motor tasks, the correlation matrices obtained for every subject and experimental condition were analyzed with the Network-Based Statistics (NBS) toolbox separately for each band. NBS is a statistical nonparametric technique that operates directly on raw connectivity values and seeks to identify potentially connected structures formed by a set of suprathreshold links (graph components; Zalesky et al., 2010). For the comparison between fixation and hand/feet movement, and between high and low performers (see next subsection), changes in graph components were tested by using a range of primary (*t*-statistic) thresholds, ranging from 5 to 9. Permutation testing (*n* = 5,000) was then used to ascribe a *p*-value. Each component identified by NBS satisfied *p* ≤ 0.05. For the graph visualization, we used the MATLAB toolbox BrainNet Viewer (Xia et al., 2013). For each band, we then counted the relative number of connections changed within the network, across networks, and for each network. Then, we analyzed possible links between the correlation changes and behavior. To this aim, according to clustering indices obtained from a *K*-means algorithm, we split our sample into two groups, comparing the task versus rest differences. Finally, we counted the number of component links modified within and across networks. We are aware that the choice of this threshold certainly influences the size of the obtained components also due to the contribution of false-positive links in a component (Zalesky et al., 2010). Nevertheless, we here computed the percentage of modified links involving each RSN, normalized by their total number to compensate for the effect of false-positive links.

#### Regression model task-rest and clustering algorithm

For each subject, band, and network, we aimed at estimating to which extent intrinsic connectivity predicted task connectivity. First, for each RSN we considered all within and across-network connections. Then, we adopted the following linear model for the task versus rest connectivity for each RSN (Tommasin et al., 2018):

T=βR+ε,

where *T* is the BLP connectivity for the motor task, *R* is the BLP connectivity during rest, *β* is the slope of the linear model, and *ε* is the error. We used all the pairwise connectivity values of each RSN to estimate *β*. This provides, for each subject and frequency band, a matrix of *β* (network × network). Specifically, *β_ij_* close to or different from 1 signaled respectively similarity or dissimilarity between rFC and tFC for the across-RSN interaction involving RSN *i* and *j*. Then, we used a data-driven approach running a *K*-means algorithm on the individual *β* matrices to cluster them (Fig. 1*D*). To estimate the optimal number of clusters, we varied the number of clusters from 2 to 10 choosing the best value as the one producing the maximum average value of the silhouette (Kaufman and Rousseeuw, 1990) provided that its values were always positive (Extended Data Fig. 3-1). We then analyzed the link between the clusters and the manual dexterity through *t* test comparisons.

#### Analysis of segregation/integration

We then investigated possible links between the individual manual skill and the task-induced modulations of the segregation/integration balance. First, the individual BLP connectivity matrices were transformed into binary graphs according to a percolation analysis (de Pasquale et al., 2021) looking for the maximum threshold ensuring the full connectedness of the graph (i.e., the number of graph components was equal to the graph size). Then, we applied Louvain modularity, as implemented in the Brain Connectivity Toolbox (Rubinov and Sporns, 2010) to estimate the global modularity for each subject and condition (rest, hand motor task). The Louvain modularity was estimated at 10,000 times for each subject, retaining the maximum modularity value together with the corresponding modules. We then applied a mixed ANOVA with condition (rest, motor task) and subject group (according to the clustering results) as factors. Duncan post hoc was applied to significant effects. In addition to segregation analysis, we also investigated how the motor task modulated the central role of networks and hubs. Thus, for each cluster, the nodal participation index (PI) was estimated over the modules in each condition. The PI values were analyzed at the RSN level, through the mean PI over the nodes in each RSN, and at the nodal level. In the first case, we analyzed possible differences in the PI across groups, conditions, and RSNs through a mixed ANOVA, with Duncan post hoc for the analysis of significant effects. Finally, at the nodal level, we ran a *t* test comparing task and rest conditions in each group (*p* < 0.05). Nodes significantly changing their PI were displayed on the cortex through BrainNet Viewer.

## Results

### Hand movements decrease the strength of functional connectivity

In analogy with previous studies (Betti et al., 2013; Cole et al., 2014; Krienen et al., 2014; Spadone et al., 2015), we found that the execution of motor tasks preserved the overall topography of MEG connectivity across all RSNs. The observed similarity (Mantel test, *p* < 0.05, *r* > 0.83) between rest and motor tasks indicates a similar functional architecture across conditions (Extended Data Fig. 2-1). Next, we investigated the influence of movements (hand, foot) on intrinsic FC (rest). Since all subjects were right-handed, we focused our analysis on the right hand, considering the left hand as a control.

**Figure 2.**
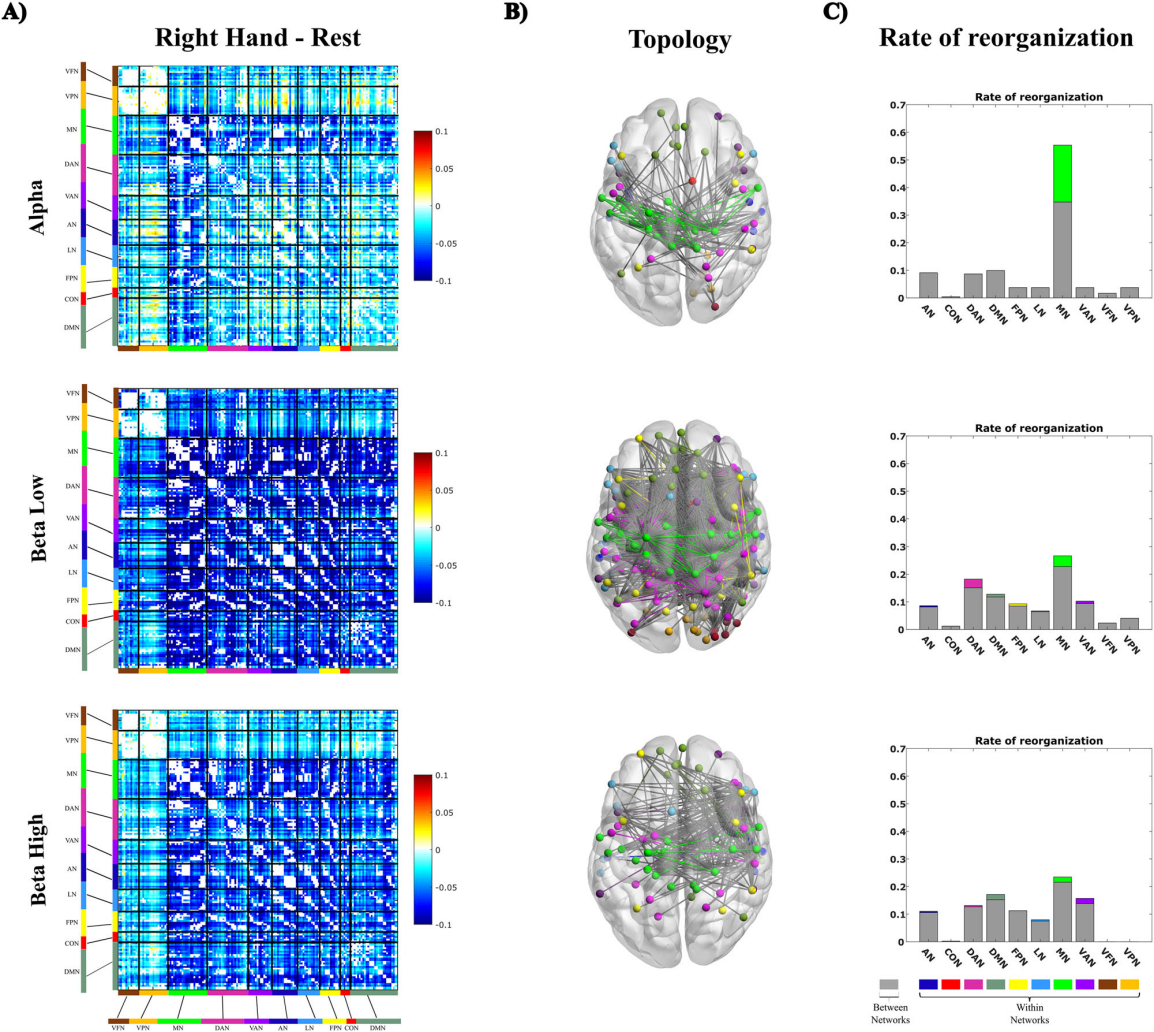
Changes of functional connectivity and topology induced by the finger tapping with the right hand. ***A***, Group-level difference connectivity matrices task-rest for α, low β, and high β bands. For visualization purposes, the matrices depict node-to-node correlation values. ***B***, changes of network topology. Right-hand movements modulate fewer links in the α band, especially within the motor network (green edges). Conversely, in the β band, we observed a widespread topological reorganization across all networks. ***C***, Percentage of modulated links. Within-network connections are color-coded, between-network connections are shown in gray. See Extended Data Figures 2-1–2-4.

We ran a repeated measures ANOVA with band (α, low β, high β), condition (right hand-rest vs foot-rest), and network (all RSNs) as within-subject factors on the connectivity modulation (task-rest) averaged across connections of each RSN. Right-hand (Fig. 2*A*) and foot movements (Extended Data Fig. 2-3*A*) induced an overall decrement of connectivity across all RSNs and bands. Specifically, we found a significant main effect of band (*F*_(2,92)_= 12.14 *p* = 0.000021, pη^2^ = 0.21) with smaller decrements in α as compared with all other bands (mean FC_α_ = −1.55, FC_low β_ = −3.73, FC_high β_ = −2.50, post hoc Duncan corrected, *p *= 0.000058 and *p* = 0.036, respectively). There was also a significant main effect of condition (*F*_(1,46)_ = 6.03, *p *= 0.018, pη^2^ = 0.12) with stronger decrements during foot as compared with right-hand movement (mean FCr_right hand_ = −2.22, mean FC_feet_ = −2.97; *p*_Bonff_ = 0.018) (Fig. 2*A* and Extended Data Fig. 2-3*A*). Furthermore, we found a significant effect of network (*F*_(9,414)_ = 11.77, *p *= 1.1 × 10^−16^, pη^2^ = 0.20) as well as a band–network interaction (*F*_(18,828)_ = 7.25, *p *< 0.001, pη^2^ = 0.14) due to a weaker decrement in the motor network in the α band (FC_α MN_ = −1.29) as compared with the low β (FC_low β MN_ = −3.69; *p*_Bonff _= 0.00001) and high β bands (FC_high β MN_ = −2.67; *p*_Bonff _= 0.000017). Finally, all the other interactions were significant (all *p*-values <0.05) except for the band–condition interaction.

10.1523/JNEUROSCI.1766-23.2024.f2-1 Figure 2-1 **Large-scale topography of connections is preserved across motor tasks and bands.** The high correlation (r > 0.83) across conditions and bands suggests that the overall topography is maintained between rest and task. See Figure 2. Download Figure 2-1, TIF file.

10.1523/JNEUROSCI.1766-23.2024.f2-2 Figure 2-2 **Changes of functional connectivity and topology induced by performing finger tapping with the left hand. A)** Group level difference connectivity matrices task-rest for alpha, beta low and beta high bands. Here depicted node to node correlation values, **B)** Changes of network topology. Left hand movements induce a similar modulation of links as the one induced by the right hand: in alpha we can observe fewer modulations circumscribed to the motor network, conversely in the beta bands there is a wide-spread reorganization **C)** Percentage of modulated links. Within-network connections are color-coded, between-network connections are shown in grey. See Figure 2. Download Figure 2-2, TIF file.

**Figure 3.**
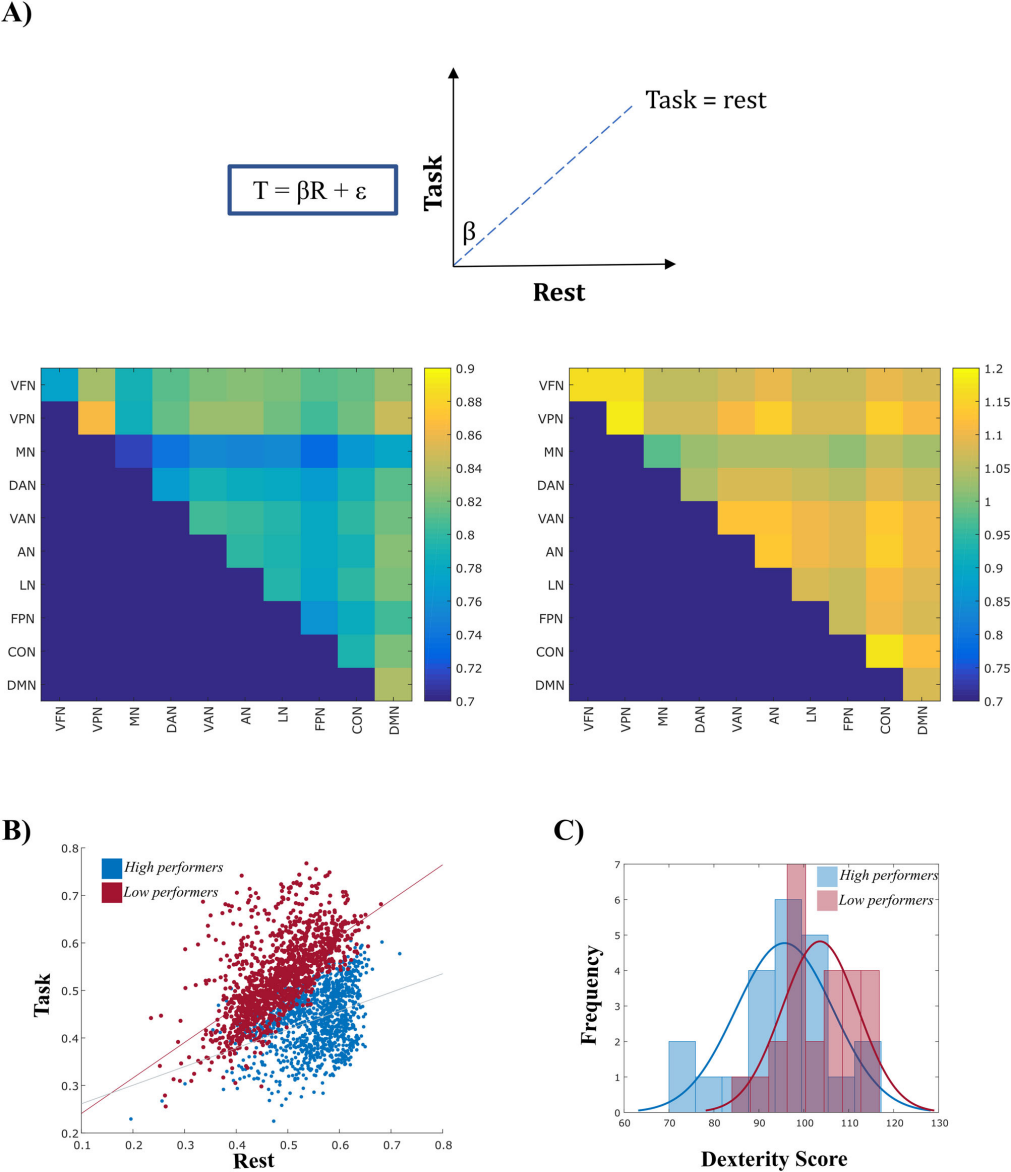
*K*-means clustering identifies high and low performers. ***A***, Centroids of the two clusters, consisting of *β* values, identified by *K*-means. ***B*** Scatterplot depicting the linear relationship between rest and task connectivity in the two groups (blue, high performers; red, low performers). A distinct linear trend is evident. ***C***, The two groups differed in terms of manual dexterity. The dexterity score was measured as mean RTs on the nine-hole peg test (see Materials and Methods). The histogram for the dexterity scores is reported for high land low performers*.* See Extended Data Figure 3-1.

10.1523/JNEUROSCI.1766-23.2024.f2-3 Figure 2-3 **Foot movements decrease the overall connectivity. A)** A widespread decrease of functional connectivity during toe squeezing in all bands is observed. The decrease during foot movements is higher than during hand movements. For visualization purposes the matrices depict node to node correlation values. **B)** Network Based Statistics analyses show that foot movements lead to an overall brain reorganization larger than with hand movements. **C)** Rate of reorganization in the three frequency bands. Within network connections are color-coded. See Figure 2. Download Figure 2-3, TIF file.

**Figure 4.**
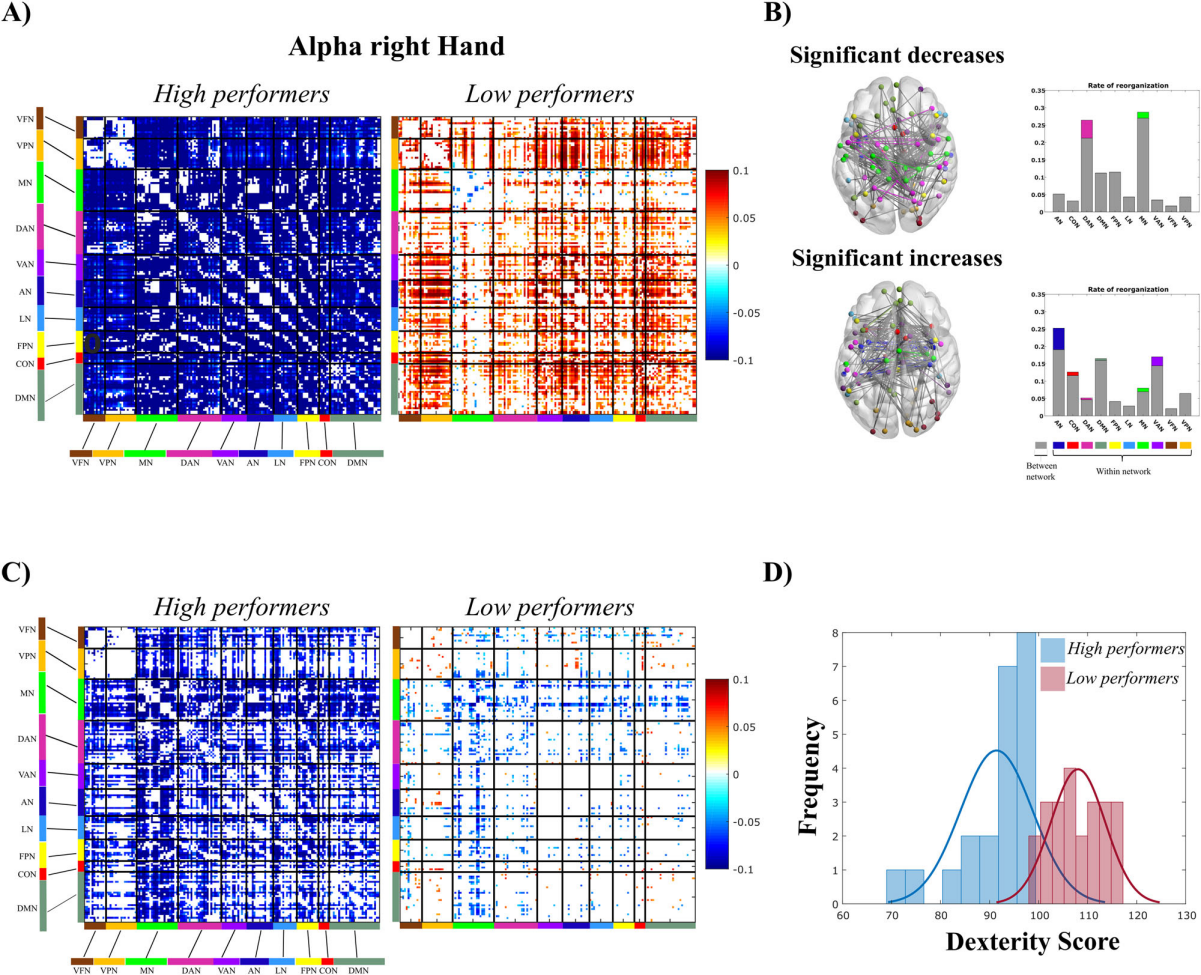
From brain-to-behavior and behavior-to-brain approach: the modulation of functional connectivity encodes manual dexterity. ***A***, Difference (task-rest) connectivity matrices for high performers (left panel) and low performers (right panel). Only significant connections are reported (*t* test). High performers exhibit an overall decrease of FC in all networks, while low performers show a slight increase, with stability in the motor network. ***B***, Modulation of topology in high and low performers and rate of reorganization expressed as a percentage of modulated links. Within-network connections are colored-coded, and between-network connections are shown in gray. ***C***, As in panels ***A*** and ***B***, we report the difference (task-rest) connectivity matrices for the high performers (left panel) and low performers (right panel) identified through a median split on the behavioral performance (nine-hole peg test). According to the results extracted through the *K*-means, high performers exhibit a decrease in FC, while low performers show similarity between rest and task matrices. ***D***, Histogram for the dexterity scores for high land low performers, according to the median split procedure. See Extended Data Figure 4-1.

10.1523/JNEUROSCI.1766-23.2024.f2-4 Figure 2-4 **Foot movements reorganize topology differently than hand movements.** Toe squeezing induces a higher modulation of links than finger tapping, but notably the reorganization involves less the motor network. See Figure 2. Download Figure 2-4, TIF file.

As a control analysis, we ran the same repeated measures ANOVA on the task performed with the left hand (Extended Data Fig. 2-2*A*). Again, we found a significant main effect of band (*F*_(2,92)_ = 13.44 *p* = 0.000008, pη^2^ = 0.23), with α connectivity values higher than all other bands (mean FC_α_ = −1.42, FC_low β_ = −3.88, FC_high β_ = −2.73, post hoc Duncan corrected, *p *= 0.00005 and *p* = 0.007, respectively). We found a trend for the main effect of condition (*F*_(1,46)_ = 3.97, *p* = 0.05) with decrements during feet movements slightly higher compared with left-hand movements (mean FC_left hand_ = −2.39, mean FC_feet_ = −2.97).

Once again, we found a significant band–network interaction (*F*_(18,828)_ = 6.89, *p = *2.2 × 10^−16^, pη^2^ = 0.13) due to a weaker decrement in the motor network in the α band (FC_αMN_ = −1.18) as compared with the low β (FC_low β MN_ = −3.86) (*p*_Bonff _= 0.00001) and high β bands (FC_high β MN_ = −2.98; *p*_Bonff _= 5 × 10^−9^). All other interactions were significant except for band–condition and condition–network interactions (all *p*-values < 0.05).

To summarize, in agreement with previous MEG reports on visual stimuli (Betti et al., 2013, 2018), all RSNs decreased their connectivity during task (motor) performance, despite maintenance of the overall resting state topography. Functional connectivity decrements spread along the entire cortical mantle and were stronger for the foot than hand movements. Interestingly, α band decrements were less prominent than in other bands, especially in the motor network.

### Reorganization of network topology during finger tapping

Next, we investigated changes in functional topology induced by the two motor tasks by means of NBS (Zalesky et al., 2010). Figure 2*B* shows the graph components that significantly decreased for each frequency band for the right-hand movement (see Extended Data Fig. 2-2*B* for the left hand). In the α band, the task produced a high proportion of decreased connections (*n* = 121), especially in the motor network (namely 23%, computed as the ratio between the number of changed connections within the motor network divided by the total number of changed connections; Fig. 2*B*, upper panel, green lines). Differently, in the low β band, while a larger number of connections (namely 1,303, approximately one order of magnitude larger than in the α band) were also reduced, we did not observe a predominant proportion involving the motor network (only 8%; Fig. 2*B*, middle panel). The same applies to the high β band, where we observed an intermediate number of decreased connections (namely 213; Fig. 2*B*, lower panel). In this case, the percentage contribution within the motor network was 4%. To illustrate the contribution of each RSN, Figure 2*C* shows the between- (gray) and within-network (color-coded) connections. It can be noted that, only in the α band, do we observe a predominant contribution within connections of the motor networks. In the other bands, changes were more uniformly distributed across RSNs.

As a control, we performed, separately, the same analyses for toe squeezing and finger tapping performed with the left hand. The results on the left hand mirrored the ones found on the right hand, supporting a larger modulation within the motor network in the α band (Extended Data Fig. 2-2*B***,***C*). As before, in the β bands (Extended Data Fig. 2-2*C*, middle and lower panels), we found a modulation of network topology involving all RSNs.

In general, foot movements reorganized many connections in all bands (Extended Data Fig. 2-3). This was further corroborated by NBS analysis comparing the two motor tasks with rest (hand-rest vs foot-rest). Among these decreased connections, the vast majority involved connections between networks with a small contribution of links within the motor network (Extended Data Fig. 2-4). In summary, motor tasks induce an extensive topological reorganization in both β bands. For the hand movement, it involved predominantly the motor network in the α band.

### The modulation of α band connectivity encodes manual dexterity

To test the behavioral relevance of intrinsic connectivity modulation in the motor cortex, we tested its relationship to manual dexterity, as measured by the nine-hole peg test.

First, to evaluate the similarity between FC at rest (rFC) and during the task (tFC), we adopted a linear model: *tFC = β × rFC + e* (Fig. 1*D*), and we studied the variations of *β* values (i.e., the slope). Specifically, for every RSN, we considered its set of connections (both within and across RSNs), and from them, we estimated the *β* values. This provides, for each subject and frequency band, a matrix of *β*, with *β_ij_* representing the tFC–rFC relationship between networks *i* and *j*. Specifically, the larger the distance of *β_ij_* from one, the higher the change from rFC to tFC. Hence, for each participant, we obtained a specific profile of functional reorganization between rest and task. Then, subjects were clustered based on their β profiles using a *K*-means clustering. We selected the number of clusters based on the maximum average value of the silhouette (Kaufman and Rousseeuw, 1990). For the α band, the optimal number of clusters was two (Extended Data Fig. 3-1).

We then analyzed the link between the clusters and the manual dexterity through *t* test comparisons.

The centroids of the obtained clusters, that is, the average of the *β* matrices within each class, are shown in Figure 3*A*. In the first group (Fig. 3*A*, left panel), *β* values were smaller than 1 (mean value = 0.77), suggesting changes in the considered connections between task and rest. By contrast, in the other cluster (Fig. 3*A*, right panel), *β* values were closer to 1 (mean value = 1.16), suggesting a higher similarity between rFC and tFC. This different trend in the two groups is evident in Figure 3*B*, where we report the complete β pattern for each subject in the two groups. Then, we tested whether the two groups reflected a different behavioral performance, as investigated through the manual dexterity task (i.e., the nine-hole peg test). Interestingly, we obtained a significant difference in terms of dexterity (*t*_(45)_ = −2.80, *p *= 0.008). The first group was characterized by faster reaction times (mean RT ± SD = 95.70 ± 10.85 s; i.e., high performers), while the second one showed slower reaction times (mean RT ± SD = 103.60 ± 8.48 s; i.e., low performers; Fig. 3*C*). Of note, these differences cannot be ascribed to either demographic or cognitive differences, as reported in Extended Data Table 3-2 (upper part). Specifically, the cognitive differences were tested through a Flanker test, provided by the HCP database. Notably, this result was specific to the α band. We did not obtain any significant clustering either in low or high β bands (Extended Data Table 3-1).

10.1523/JNEUROSCI.1766-23.2024.f3-1 Figure 3-1 **Estimation of the optimal number of K-means classes.** Silhouette as a function of the number of classes in the alpha band for the right hand. Corresponding at 2 clusters we obtained the highest average silhouette, with no negative values. This value was adopted as the optimal number of K-means clusters. See Figure 3. Download Figure 3-1, TIF file.

10.1523/JNEUROSCI.1766-23.2024.t3-1 Table 3-1 **Clustering is effective only in the alpha band.** The numerosity of the classes obtained by K-means at different percentiles (see Materials and Methods) in the alpha and beta bands. No significant effects were obtained in the beta low and beta high, while in alpha, the clustering successfully identified *High* vs *Low* performers at percentiles lower or equal to 85. Download Table 3-1, DOCX file.

10.1523/JNEUROSCI.1766-23.2024.t3-2 Table 3-2 **Demographics and cognitive characteristics** of *High* and *Low* performers according with k-means (upper part) or median-split (bottom-part). Mean and (standard deviation) are shown. Download Table 3-2, DOCX file.

In the α band, we then tested the stability of the *β* values and the related clustering when pruning, at increasing levels, the original set of connections, to assess which was the range of connections mainly driving the classification. To do so, we thresholded the connectivity matrices preserving only connections above an *N* percentile value (Extended Data Table 3-1), from *N* = 0 to *N* = 0.99. We then computed *β* values again for each network subset and we repeated *K*-means clustering. Our results demonstrate that the algorithm separates two groups, only for connections not exceeding the 85th percentile. The two groups could not be separated when looking at the strongest connections (>85th percentile). However, the characterization of these connections is beyond the scope of this paper. The analysis shown in Extended Data Table 3-1 suggests that the clustering results are consistent and reproducible with respect to the choice of the adopted thresholds. Notably, the same analyses did not produce any consistent results either in low β or high β frequency bands.

In summary, this analysis shows that the modulation of functional connectivity, in the α band, encodes the behavioral performance.

### Differences in brain connectivity between high and low dexterity participants

Figure 4*A* shows the different patterns of FC modulation in going from rest to task (finger tapping) in high and low performers. High performers show an overall significant decrease in connectivity across all RSNs (Fig. 4*A*, left). Conversely, low performers display a generally significant increase in FC strength involving all RSNs, apart from MN and DAN (Fig. 4*A*, right). We tested, separately for the two groups, which graph components were modulated by finger tapping. As shown in Figure 4*B* (upper panel), participants with higher dexterity showed significant decrements of correlation mainly between networks. On the contrary, low performers showed significant increments of correlation, both between- and within-network (lower panel).

10.1523/JNEUROSCI.1766-23.2024.f4-1 Figure 4-1 **Connectivity modulations are band-specific in the behaviour-to-brain approach. A)** *High* and *Low performers* show a different modulation of task-rest FC specifically in the alpha band (upper panel). This difference was statistically significant according to a t-test (alpha =0.05, two-tailed test). **B)** Number of connection changes between the two groups in each network. Crucially, in the alpha band we observe a larger difference between *High* and *Low performers.* See Figure 4. Download Figure 4-1, TIF file.

To further validate this relationship between modulations of FC and dexterity (from brain to behavior), we first divided participants based on their performance, and then we analyzed the connectivity modulations (Fig. 4*C*; from behavior to brain). We adopted a median split approach to obtain high and low performers (based on RTs on the nine-hole peg test), as shown in Figure 4*D*. Then, as in the previous analysis (Fig. 4*A*), we compared the FC modulations within these two groups. In agreement with the first analysis, high performers show large FC decrements across all RSNs, differently from low performers characterized by smaller FC changes (Fig. 4*C*). Again, these differences cannot be ascribed to either demographic or cognitive differences, as reported in Table 3-2.

The brain-to-behavior approach shows a specificity attributed to the α band (as revealed through the clustering approach). To test this specificity also in the behavior-to-brain approach, we considered the division of high and low performers obtained from the median split. Then, we tested the similarity of their connectivity modulation matrices in the α, low β, and high β bands, across subjects through an unpaired *t* test (*α* = 0.05, two-tailed test). In the α band, we obtained that a large number of connectivity changes resulted in a significant difference between the two groups. Conversely, both in the low and high β, this number was highly reduced (∼40% of the connection changes observed in α). This suggests a higher similarity in the two groups in the β as compared with the α band. In this band, in the high performers, we observed an overall reorganization involving all RSNs. This pattern was completely different in the β bands, where we observed changes involving only VFN, VPN, and DAN networks (Extended Data Fig. 4-1). This seems to suggest a specificity for the α band and also for the behavior-to-brain approach.

It must be considered that these analyses address the connectivity–behavior link from two opposite perspectives: from the FC to behavior and the other way around. Thus, in the first case, the connectivity modulations in the two groups are naturally enhanced, being optimized by the clustering, while the resulting group division is somehow smoother. In the second analysis, instead, the groups are more clearly distinguished, being separated by the median split, while the corresponding differences in FC modulations are more moderate than in the previous case. However, it is noteworthy that in both analyses the results are consistent in showing opposite FC patterns between groups.

### Task-induced modulations of segregation/integration reflect subjects' dexterity

**Figure 5.**
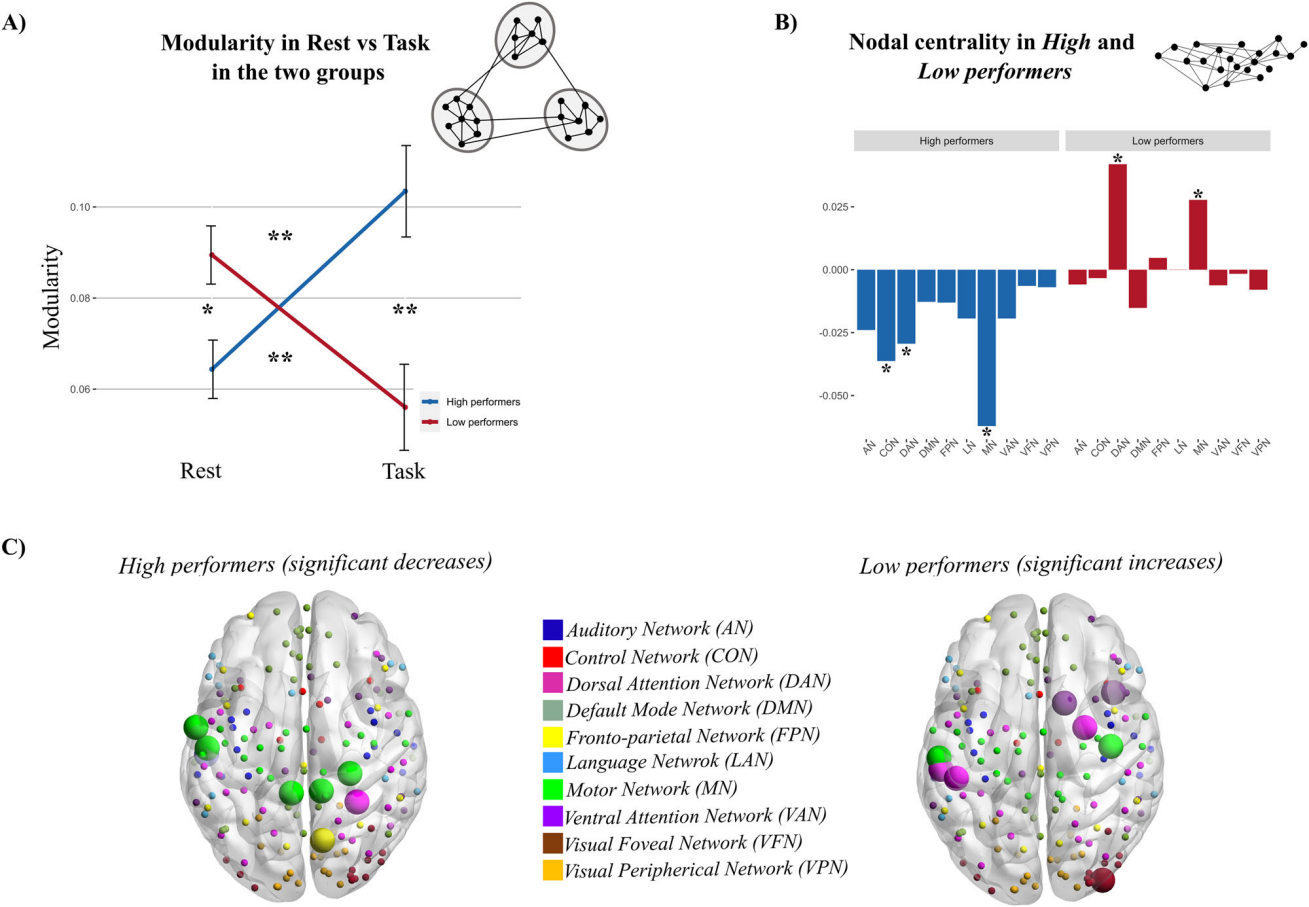
Functional integration and segregation in high/low performers. ***A***, High performers exhibit a statistically significant higher modularity during finger tapping than rest (*p < 0.05, **p < 0.01, ***p < 0.001); conversely, modularity decreases in low performers when switching from rest to motor task. The different modularity in the two groups suggests that segregation relates to the performance. ***B***, The PI shows a decrease of nodal centrality (averaged over nodes in each RSN) in high performers, especially in the motor, control network, and DAN. Conversely, in low performers, the PI increases. This modulation is significant in the motor network and control network. ***C***, Nodes with significant decreases in PI between rest and task in high (left) and increases in PI in low performers (right). Here, the differences in PI between task and rest for each RSN are depicted for visualization purposes. The statistically significant nodes are enlarged in the figure.

Our previous findings show that specific patterns of FC modulation from rest to movement depend on the participants' motor skills (i.e., performance on the nine-hole peg task). Based on past evidence of motor learning inducing increased segregation of the sensorimotor system and reduction of hub centrality (Bassett et al., 2015), we estimated graph modularity in the α band by quantifying the amount of segregation. We first applied a percolation analysis (Bordier et al., 2017) to obtain individual binary graphs. Then, we applied the Louvain modularity (Lancichinetti and Fortunato, 2009) and ran a mixed model ANOVA on modularity, with group (high and low performers) as the between-subject factor and experimental condition (rest, hand motor task) as the within-subject factor. We found a significant group–condition interaction (*F*_(1,45)_ = 20.46, *p *= 0.00004, pη^2^ = 0.31) explained by an increase of network modularity when going from rest (fixation) to hand movements (finger tapping) in high performers (*p *= 0.002) and a decrease of modularity in low performers (*p *= 0.007). Moreover, modularity in high performers was significantly smaller than that in low performers at rest (*p* = 0.03), while it became larger than that in low performers during finger tapping (*p *= 0.0002; Fig. 5*A*).

Then, we measured modulations of centrality through the PI (de Pasquale et al., 2018). The results obtained from a mixed model ANOVA, with group (high and low performers) as a categorical factor and experimental condition (rest, finger tapping) and RSNs (all networks) as within-subject factors, revealed a significant condition–group–RSN interaction (*F*_(9,405)_ = 2.84, *p *= 0.003, pη^2^ = 0.06). When going from rest to task, high performers showed a significant decrease of the PI in the control network (CON; *p *= 0.02), DAN (*p* = 0.04), and MN (*p *= 0.000007). Conversely, low performers showed a significant increase in DAN (*p *= 0.003) and MN (*p *= 0.04). For the sake of clarity, Figure 5*B* shows the difference between task and rest in the two groups. At the level of individual regions (see Materials and Methods), high performers showed a significant decrease of centrality in nodes of the MN (dmSPL, vCS, S2, dPoCe, mdSPL), FPN (PrCu), DAN (mIPS), and AN (mSTG). Conversely, low performers showed significant PI increases in the MN (vPoCe, lvPoCe), DAN (vPoCe-SMG, dPoCe, FEF), VAN (vPrCe, MFC2), and VFN (LO; Fig. 5*C*).

In summary, when executing the finger tapping task, dexterous individuals show an increase in the segregation of the intrinsic network topology. In parallel, they show a reduction of centrality, mainly in the MN, DAN, and CON. The reduction of PI indicates that, during the finger tapping task, the connections underlying the hub centrality involve more within-network connections than across-network ones. This suggests a “focusing” effect of the involved networks that reduces their communication with other RSNs. Opposite patterns were found in low performers, with an increase in across-network communication mainly involving the DAN and MN.

## Discussion

Manual dexterity is a long-term skill that varies among individuals, and this study investigated how this characteristic is reflected in the task-induced modulation of spontaneous brain connectivity. We confirmed that the underlying network topography observed at rest closely resembled that observed during a motor task. However, in the α and β frequency bands, this stable topography is combined with consistent changes in connectivity strength and topology. Notably, in the α band, a specific reorganization of connections enabled the differentiation of high performers from low performers. This reorganization manifested in opposite directions for the two groups: high performers displayed primarily decreased motor network connections, while low performers exhibited more widespread changes that extended beyond the motor network, with slight increases or greater stability. Notably, high performers demonstrated an “internal focusing” effect in network topology. This was characterized by increased network modularity, indicating enhanced segregation, and decreased nodal centrality. Conversely, low performers displayed an opposite trend, suggesting a dysfunctional integration.

Proficiency in using hands is revealed through specific modulations of functional architecture in the α band. The functional significance of this rhythm is multifaceted and under debate. Traditionally, its amplitude has been associated with the inhibition of task-irrelevant regions in the brain (Klimesch et al., 2007; Jensen and Mazaheri, 2010). α is an “idling” rhythm (Pfurtscheller et al., 1996), that is, denoting a state of inactivity of the brain circuits, which is then desynchronized during a task. The α connectivity needs to be suppressed because more specific task-related functional patterns emerge (Betti et al., 2013, 2018). Such an effect (Fig. 2, Extended Data Figs. 2-2, 2-3) is also in line with decrements of correlated cortical noise occurring during tasks or stimulus presentation, observed in the monkey and cat visual cortex (Smith and Kohn, 2008; Nauhaus et al., 2012). These α oscillations have been also linked with high-order cognitive functions, such as memory and attention (Klimesch, 2012; Sadaghiani and Kleinschmidt, 2016). They correlate with faster reaction times, better memory performance, and information processing (Klimesch et al., 1993, 1996; Angelakis et al., 2004). A link between α rhythm and behavior has been recently highlighted during resting state, particularly in expert populations. In a sample of pianists, the spontaneous phase coupling in the α band correlates with the motor performance of finger tapping (Allaman et al., 2020). Analogously, in expert dancers, resting state connectivity in the μ rhythm, successfully decodes the level of motor expertise (Amoruso et al., 2017, 2022). Accordingly, motor (Albert et al., 2009; Tambini et al., 2010; Ma et al., 2011; Taubert et al., 2011; Gabitov et al., 2019) and perceptual learning (Lewis et al., 2009) shapes intrinsic connectivity in a behaviorally relevant manner. In addition, resting state cortical connectivity predicts future motor skill and visual perceptual learning acquisition (Baldassarre et al., 2012; Wu et al., 2014; Dyck et al., 2021) and interindividual variability in motor performance (Roshchupkina et al., 2022).

Our findings add novel information to this body of work.

In fact, although theoretical models predict that spontaneous connectivity reflects the training of cortical networks in the course of development, first, and then daily life, most of the experiments performed to date adopted laboratory tasks (Harmelech and Malach, 2013; Betti et al., 2021; Pezzulo et al., 2021). Here, we considered a finger tapping task, a controlled movement that closely resembles the precision grip thumb index (NAPIER, 1956; Castiello, 2005), used frequently and consistently by people in daily life (Sili et al., 2023). Hence, one might argue that this movement, frequently repeated during grasping of small objects, may be stored in patterns of intrinsic connectivity within the motor cortex. We show that finger tapping differentially modulates α, not β, band connectivity in the motor cortex, especially for participants displaying stronger manual dexterity in the nine-hole peg task. There are two specific modulations. Firstly, higher dexterity participants showed regionally specific decrements in the α band, especially in the motor network. Moreover, the connectivity changes were more focal and segregated. Instead, lower dexterity individuals showed no changes in the motor network and increased connectivity with more integration across networks. Secondly, toe squeezing, a movement less frequently performed in daily life, caused a global and widespread reduction of the intrinsic α connectivity across all networks.

The widespread connectivity decrements in α caused by the less practiced toe squeezing nicely dovetails with our previous work in the visual system where we observed that natural stimuli produced specific decrements in connectivity and temporal patterns of BLP correlation, which were more limited and more similar to resting state topography, and dynamics than widespread changes produced by synthetic temporally scrambled movies (Betti et al., 2018). The stronger similarity between spontaneous dynamics and dynamics produced by natural stimuli versus synthetic stimuli has been also shown in single-unit work in monkeys and ferrets (Fiser et al., 2004; Berkes et al., 2011). We have argued that this similarity reflects the function of spontaneous activity as a spatiotemporal generative model of the environment, body, and cognition (Pezzulo et al., 2021).

In contrast to α, β connectivity seems to be related to the movement itself, thus being highly modulated during both motor tasks, without specific topographic changes (Fig. 2 and Extended Data Fig. 2-3). Being β the default rhythm of the motor system, task-induced modulations of connectivity may reverberate even transiently after the task and correlate with motor learning (Mary et al., 2017). However, such an effect is not shown here, as our study is not properly designed to explore the short-term training effects.

In addition, we show that the level of long-term expertise in manual dexterity biases how the motor network responds to a common motor task. Interestingly, higher-performance individuals show more segregation while lower-performance individuals show more integration. This result has been replicated in healthy subjects where stronger network segregation predicts higher cognitive, behavior, and health performance (Smith et al., 2015) and in stroke patients where lesions cause abnormalities of cognitive functions that linearly relate to the degree of modularity and recover proportionally to its improvement (Corbetta et al., 2018).

Previous studies showed that network segregation and integration mechanisms have been associated with behavioral performance (Fornito et al., 2012; Cohen and D’Esposito, 2016; Wang et al., 2021). In our study, in high performers, the required segregation seems to steer the system toward an “internal focusing” of task-related networks. In fact, the segregation is observed through an increase in functional modularity, going from rest to task. This agrees with previous studies where modularity has been reported as sensitive to individual differences occurring during motor training (Bassett et al., 2011; Baniqued et al., 2019). An increase in modularity during active motor behavior may represent a strategy for responding more efficiently to the task demand. In fact, learned procedures are automated (Milton et al., 2004) and characterized by focal brain activity (Pascual-Leone et al., 1995; Krings et al., 2000; Kelly and Garavan, 2005). Specifically, we observed an increase in modularity achieved through an important switch of the participation indices of functional hubs (Fig. 5). In high performers, hubs of the motor and control networks showed a significant reduction of their PI when switching from rest to task. The observed trend in the PI suggests a shift in the role of the involved hubs from linking nodes of distinct communities to linking nodes within the same community. This seems to realize the above-mentioned “internal focusing” of these RSNs. This is in line with previous studies (Shine et al., 2016). As far as it regards low performers, we observed an opposite reorganization of the functional architecture, characterized by dysfunctional higher integration. Interestingly, the loss of network segregation has been reported as a dysfunctional mechanism in many disconnection syndromes, such as stroke (Spadone et al., 2022).

In summary, through the lifespan, learning processes and experience build an intrinsic scaffold of communication that needs to be stable to store procedural and semantic memories. However, in the presence of a task, it must also be flexible to enhance and support the performance. Our study suggests that these properties are embedded in the topography and topology of the modulation of the intrinsic connectivity in the α band. Jointly these observations suggest the intriguing possibility that long-term priors may be coded in α connectivity, while short-term learning in β connectivity. This hypothesis shall be tested in future experiments. Taken together, these results suggest an intriguing novel role of the functional connectivity in the α band and its relationship with behavior, well beyond the typically reported simple suppression.

Overall, our results pave the way for the development of novel and personalized therapeutic strategies for restoring pathophysiological mechanisms arising from an impairment in the coordination of distributed neural activity. This applies to a large variety of pathological disorders ranging from traumatic and vascular lesions to neurodegenerative diseases (Hohenfeld et al., 2018). For example, it has already been shown that stroke patients exhibit dysfunctional intra- and interhemispheric connectivity patterns, and this impairment in the balance of communication can predict behavioral impairment (Corbetta et al., 2018; Siegel et al., 2018). In this perspective, our results are particularly relevant, as we provide evidence that dexterity, transversal to various motor competencies, is already sculpted in neural communication. Intervening in these neural communication patterns can allow us to develop recovery strategies with potential positive impacts on daily living activities.
