# Supplementary figures and images for: Encoding Manual Dexterity through Modulation of Intrinsic α Band Connectivity
Source: J Neurosci. 2024 Mar 27;44(20):e1766232024. doi: 10.1523/JNEUROSCI.1766-23.2024 (PMC11097277; doi:10.1523/JNEUROSCI.1766-23.2024)

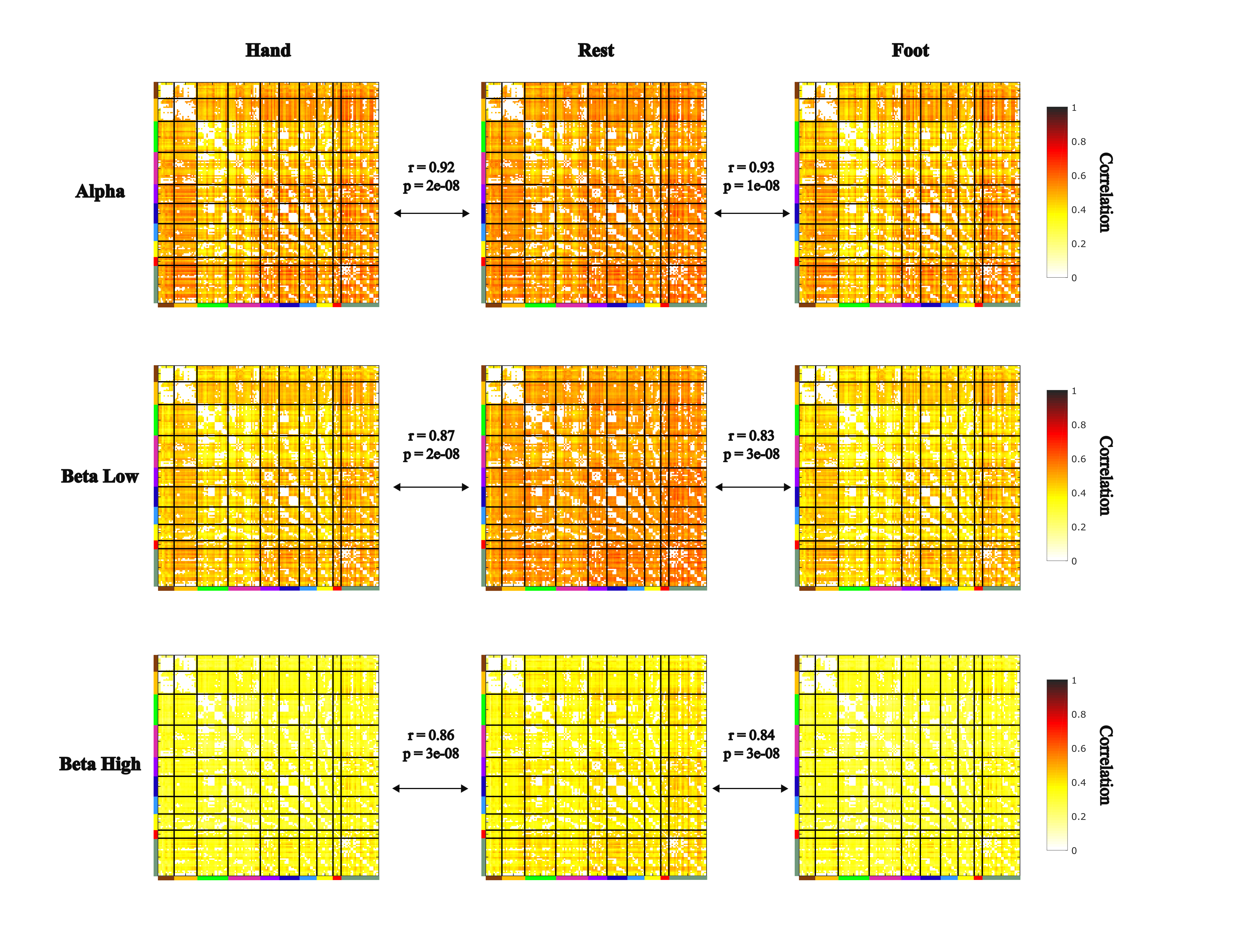

Supplement: Figure 2-1 — Large-scale topography of connections is preserved across motor tasks and bands. The high correlation (r > 0.83) across conditions and bands suggests that the overall topography is maintained between rest and task. See Figure 2. Download Figure 2-1, TIF file. [file jneuro-44-e1766232024-s001.tif]

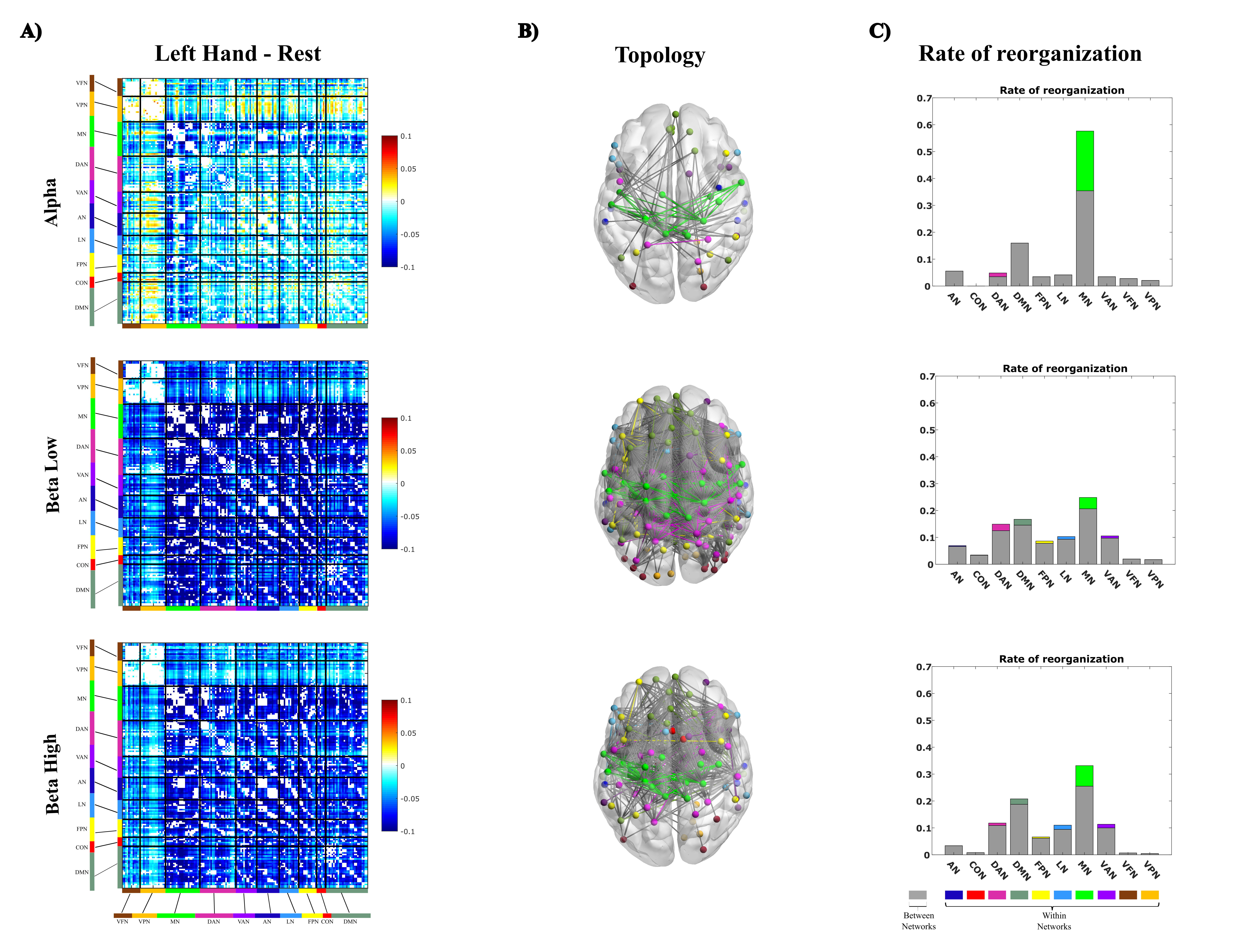

Supplement: Figure 2-2 — Changes of functional connectivity and topology induced by performing finger tapping with the left hand. A) Group level difference connectivity matrices task-rest for alpha, beta low and beta high bands. Here depicted node to node correlation values, B) Changes of network topology. Left hand movements induce a similar modulation of links as the one induced by the right hand: in alpha we can observe fewer modulations circumscribed to the motor network, conversely in the beta bands there is a wide-spread reorganization C) Percentage of modulated links. Within-network connections are color-coded, between-network connections are shown in grey. See Figure 2. Download Figure 2-2, TIF file. [file jneuro-44-e1766232024-s002.tif]

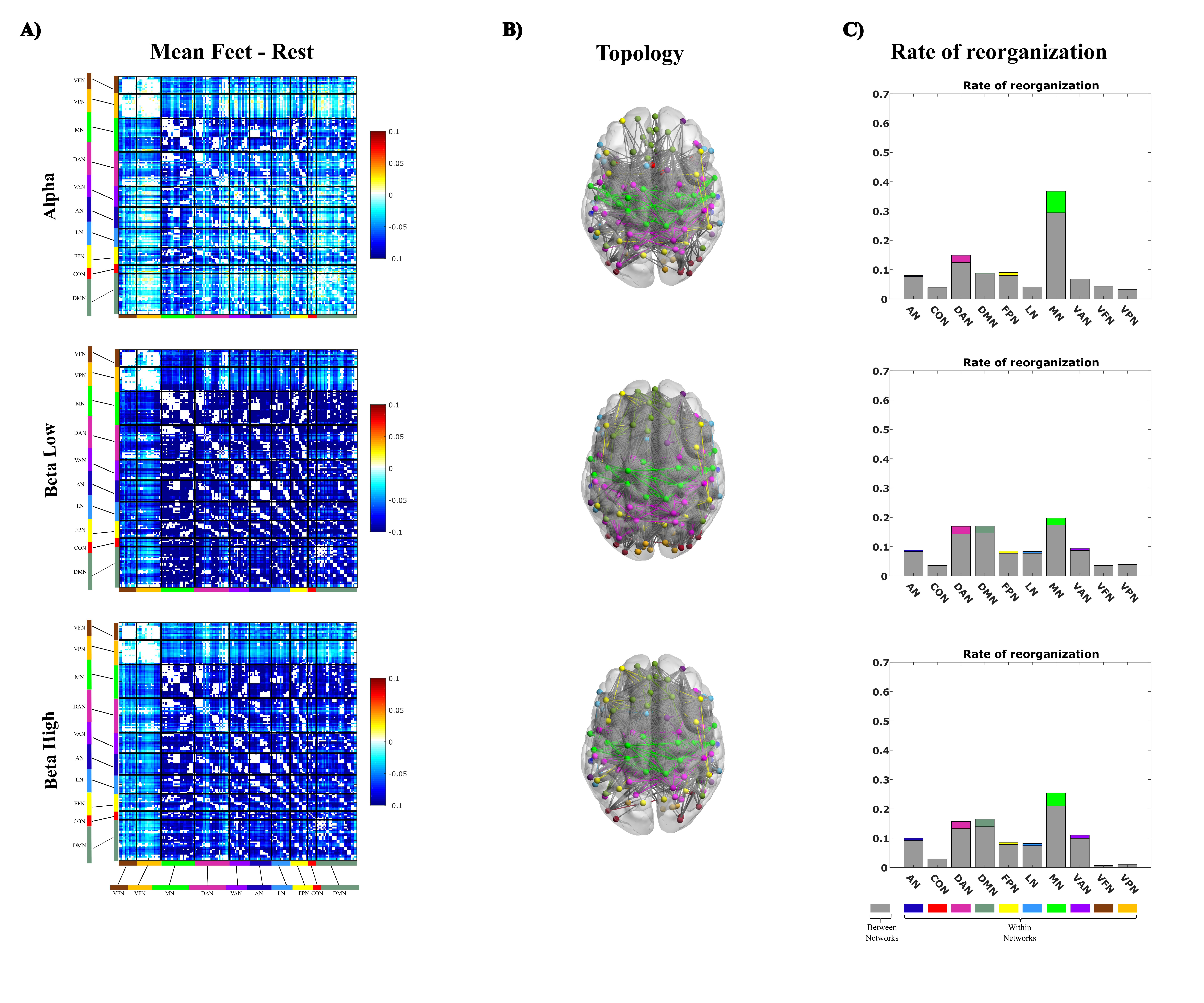

Supplement: Figure 2-3 — Foot movements decrease the overall connectivity. A) A widespread decrease of functional connectivity during toe squeezing in all bands is observed. The decrease during foot movements is higher than during hand movements. For visualization purposes the matrices depict node to node correlation values. B) Network Based Statistics analyses show that foot movements lead to an overall brain reorganization larger than with hand movements. C) Rate of reorganization in the three frequency bands. Within network connections are color-coded. See Figure 2. Download Figure 2-3, TIF file. [file jneuro-44-e1766232024-s003.tif]

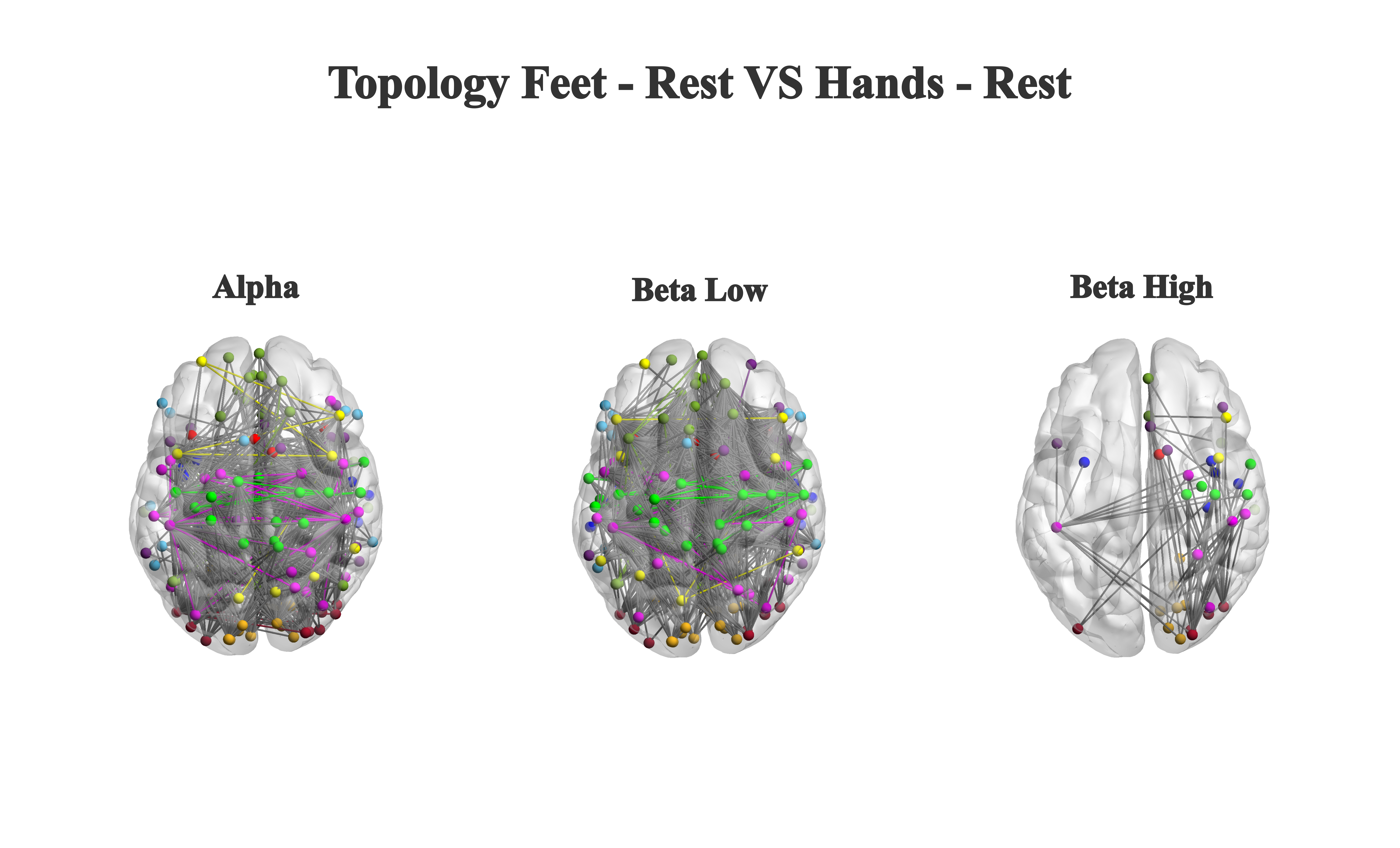

Supplement: Figure 2-4 — Foot movements reorganize topology differently than hand movements. Toe squeezing induces a higher modulation of links than finger tapping, but notably the reorganization involves less the motor network. See Figure 2. Download Figure 2-4, TIF file. [file jneuro-44-e1766232024-s004.tif]

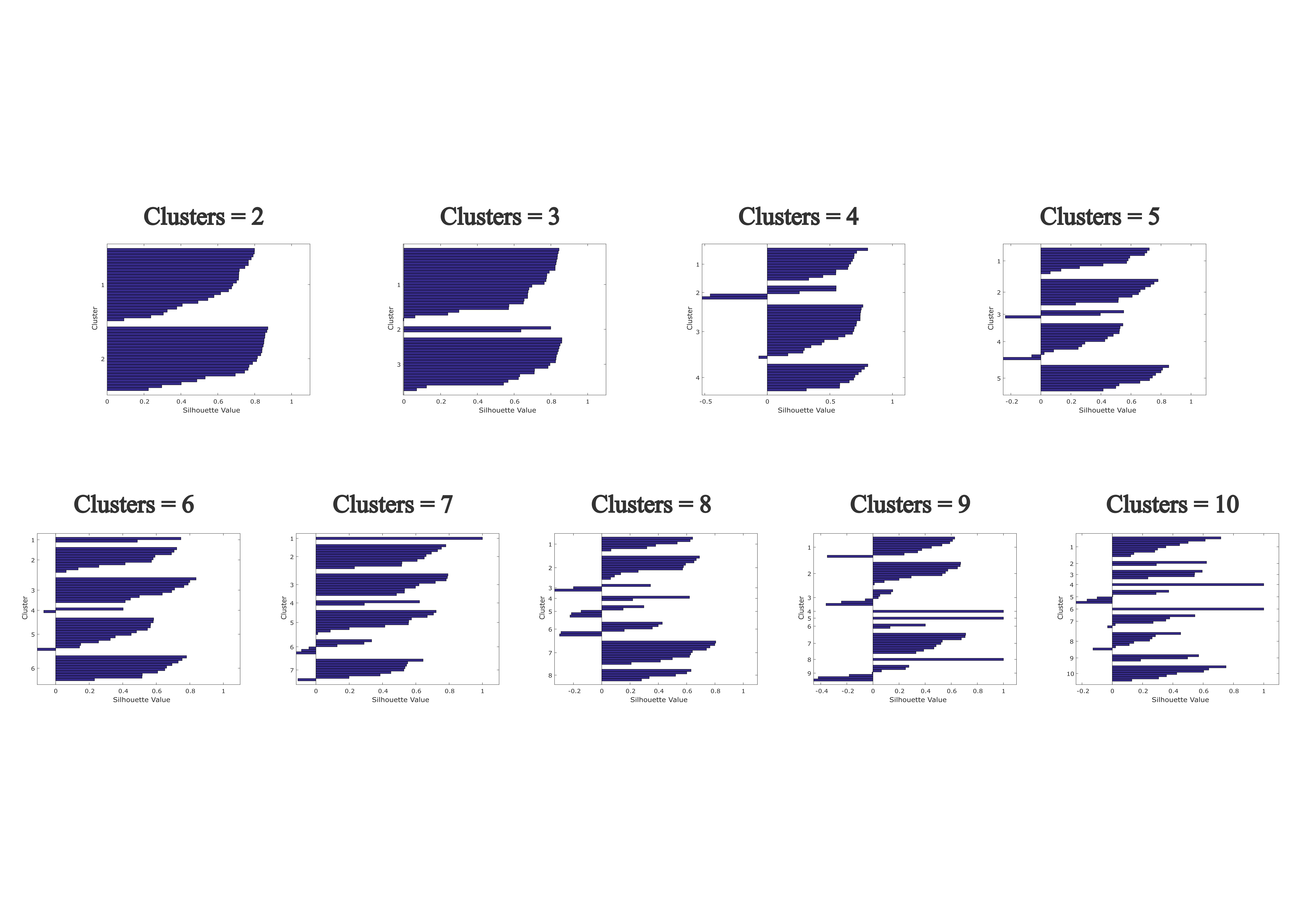

Supplement: Figure 3-1 — Estimation of the optimal number of K-means classes. Silhouette as a function of the number of classes in the alpha band for the right hand. Corresponding at 2 clusters we obtained the highest average silhouette, with no negative values. This value was adopted as the optimal number of K-means clusters. See Figure 3. Download Figure 3-1, TIF file. [file jneuro-44-e1766232024-s005.tif]

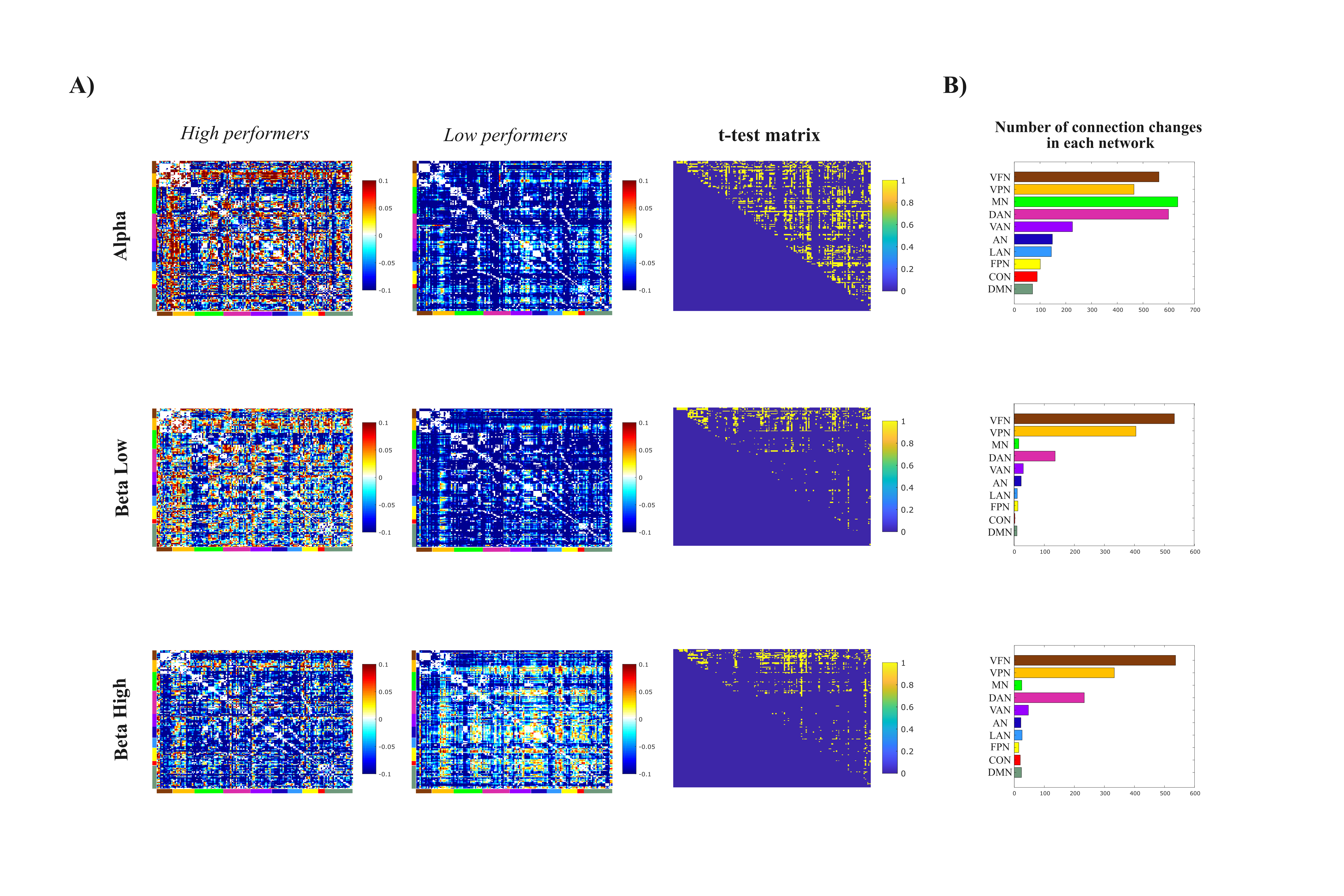

Supplement: Figure 4-1 — Connectivity modulations are band-specific in the behaviour-to-brain approach. A) High and Low performers show a different modulation of task-rest FC specifically in the alpha band (upper panel). This difference was statistically significant according to a t-test (alpha =0.05, two-tailed test). B) Number of connection changes between the two groups in each network. Crucially, in the alpha band we observe a larger difference between High and Low performers. See Figure 4. Download Figure 4-1, TIF file. [file jneuro-44-e1766232024-s006.tif]
