# Supplementary material for: Encoding Manual Dexterity through Modulation of Intrinsic α Band Connectivity
Source: J Neurosci. 2024 Mar 27;44(20):e1766232024. doi: 10.1523/JNEUROSCI.1766-23.2024 (PMC11097277; doi:10.1523/JNEUROSCI.1766-23.2024)
Supplement: Table 3-1 — Clustering is effective only in the alpha band. The numerosity of the classes obtained by K-means at different percentiles (see Materials and Methods) in the alpha and beta bands. No significant effects were obtained in the beta low and beta high, while in alpha, the clustering successfully identified High vs Low performers at percentiles lower or equal to 85. Download Table 3-1, DOCX file. [file jneuro-44-e1766232024-s007.docx]

**Table 3-1 Clustering is effective only in the alpha band.** The numerosity of the classes obtained by K-means at different percentiles (see Materials and Methods) in the alpha and beta bands. No significant effects were obtained in the beta low and beta high, while in alpha, the clustering successfully identified *High* vs *Low* performers at percentiles lower or equal to 85.

|  | **Percentile** | **Group 1 (N)** | **Group 2 (N)** | **t-test (p-value)** |
| --- | --- | --- | --- | --- |
| **Alpha** | 90 | 46 | 1 | .92 |
|  | 85 | 20 | 27 | .05 |
|  | 80 | 23 | 24 | **.02*** |
|  | 70 | 21 | 26 | **.02*** |
|  | 65 | 24 | 23 | **.005**** |
|  | 60 | 22 | 25 | **.02*** |
|  | 55 | 24 | 23 | **.03*** |
|  | 50 | 24 | 23 | **.03*** |
|  | 45 | 23 | 24 | **.03*** |
|  | 40 | 23 | 24 | **.009**** |
|  | 30 | 21 | 26 | **.007**** |
|  | 20 | 21 | 26 | **.007**** |
|  | 10 | 21 | 26 | **.007**** |
| **Beta Low** | 90 | 46 | 1 | .23 |
|  | 85 | 46 | 1 | .33 |
|  | 80 | 1 | 46 | .33 |
|  | 70 | 46 | 1 | .33 |
|  | 65 | 1 | 46 | .33 |
|  | 60 | 13 | 34 | .62 |
|  | 55 | 13 | 34 | .62 |
|  | 50 | 33 | 14 | .63 |
|  | 45 | 14 | 33 | .63 |
|  | 40 | 15 | 32 | .44 |
|  | 30 | 31 | 16 | .39 |
|  | 20 | 16 | 31 | .39 |
|  | 10 | 19 | 28 | .24 |
| **Beta High** | 90 | 1 | 46 | .33 |
|  | 85 | 46 | 1 | .33 |
|  | 80 | 46 | 1 | .33 |
|  | 70 | 1 | 46 | .33 |
|  | 65 | 1 | 46 | .33 |
|  | 60 | 1 | 46 | .33 |
|  | 55 | 1 | 46 | .33 |
|  | 50 | 1 | 46 | .33 |
|  | 45 | 1 | 46 | .33 |
|  | 40 | 46 | 1 | .33 |
|  | 30 | 1 | 46 | .33 |
|  | 20 | 1 | 46 | .33 |
|  | 10 | 1 | 46 | .33 |
| Note: * *p*<.05 ***p*<.001 | | | | |
