# Supplementary material for: Encoding Manual Dexterity through Modulation of Intrinsic α Band Connectivity
Source: J Neurosci. 2024 Mar 27;44(20):e1766232024. doi: 10.1523/JNEUROSCI.1766-23.2024 (PMC11097277; doi:10.1523/JNEUROSCI.1766-23.2024)
Supplement: Table 3-2 — Demographics and cognitive characteristics of High and Low performers according with k-means (upper part) or median-split (bottom-part). Mean and (standard deviation) are shown. Download Table 3-2, DOCX file. [file jneuro-44-e1766232024-s008.docx]

**Table 3-2 Demographics and cognitive characteristics** of *High* and *Low* performers according with k-means (upper part) or median-split (bottom-part). Mean and (standard deviation) are shown.

| **K-means clustering** |  |  |
| --- | --- | --- |
|  | *High performers* | *Low performers* |
| N | 22 | 25 |
| Age | 28.9 ± 3.2 | 26.6 ± 4.0 |
| Sex | 13 F, 9 M | 11 F, 14 M |
| Handedness | 76.8 ± 22.3 | 80.2 ± 14.7 |
| Years of Education | 14.8 ± 2.0 | 15 ± 1.5 |
| Cognitive measure (Flanker Test) | 102.6 ± 8.4 | 106.7 ± 9.5 |
|  |  |  |
| **Median split grouping** |  |  |
|  | *High performers* | *Low performers* |
| N | 22 | 23 |
| Age | 28.7 ± 3.4 | 27.2 ± 4.0 |
| Sex | 11 F, 11 M | 13 F, 10 M |
| Handedness | 78.2 ± 21.2 | 80.9 ± 16.8 |
| Years of Education | 14.4 ± 2.1 | 15.5 ± 1.0 |
| Cognitive measure (Flanker Test) | 103.9 ± 9.1 | 105.5 ± 9.2 |
|  |  |  |
